# Substrate-induced interfacial plasmonics for photovoltaic conversion

**DOI:** 10.1038/srep14497

**Published:** 2015-09-28

**Authors:** Xinxi Li, Chuancheng Jia, Bangjun Ma, Wei Wang, Zheyu Fang, Guoqing Zhang, Xuefeng Guo

**Affiliations:** 1 School of Materials and Energy, Guangdong University of Technology, Guangzhou 510006, P. R. China; 2 Center for Nanochemistry, Beijing National Laboratory for Molecular Sciences, State Key Laboratory for Structural Chemistry of Unstable and Stable Species, College of Chemistry and Molecular Engineering, Peking University, Beijing 100871, P. R. China; 3 State Key Lab for Mesoscopic Physics, School of Physics, Peking University, Beijing 100871, P. R. China; 4 Department of Materials Science and Engineering, College of Engineering, Peking University, Beijing 100871, P. R. China

## Abstract

Surface plasmon resonance (SPR) is widely used as light trapping schemes in solar cells, because it can concentrate light fields surrounding metal nanostructures and realize light management at the nanoscale. SPR in photovoltaics generally occurs at the metal/dielectric interfaces. A well-defined interface is therefore required to elucidate interfacial SPR processes. Here, we designed a photovoltaic device (PVD) with an atomically flat TiO_2_ dielectric/dye/graphene/metal nanoparticle (NP) interface for quantitatively studying the SPR enhancement of the photovoltaic conversion. Theoretical and experimental results indicated that the graphene monolayer was transparent to the electromagnetic field. This transparency led to significant substrate-induced plasmonic hybridization at the heterostructure interface. Combined with interparticle plasmonic coupling, the substrate-induced plasmonics concentrated light at the interface and enhanced the photo-excitation of dyes, thus improving the photoelectric conversion. Such a mechanistic understanding of interfacial plasmonic enhancement will further promote the development of efficient plasmon-enhanced solar cells and composite photocatalysts.

The photovoltaic conversion of solar energy into electrical power is a promising way to provide sustainable clean energy, and to overcome energy and environment issues facing human society in the 21st century^1^,^2^,^3^,^4^,^5^. To expedite the large-scale implementation of photovoltaic technology and compete with fossil fuel-based energy, the cost of photovoltaic solar cells must be reduced^6^,^7^,^8^. Light management techniques are of great importance to realize low-cost photovoltaic conversion because they can increase the absorption of photoelectric materials in solar cells, which enhances the efficiency of photoelectric conversion and thus decreases material usage^9^,^10^,^11^,^12^,^13^. A pyramidal surface texture is frequently used for light trapping in traditional thick Si solar cells^11^. High-refractive-index dielectric and semiconductor nanostructures have been developed for photon management in thin-film solar cells^9^.

Surface plasmon resonance (SPR) involves the resonance of light waves with the collective oscillation of a gas of electrons inside a metal. It can produce a strong charge displacement in metallic nanostructures, and concentrate the light field into a small space surrounding the nanostructures^14^,^15^,^16^,^17^. Therefore, SPR has been used for nanoscale light trapping in various photovoltaic devices (PVDs)^10^,^18^, including thin-film Si solar cells^19^,^20^, organic polymer solar cells^21^,^22^, dye-sensitized solar cells (DSSCs)^23^,^24^ and quantum dot photovoltaics^25^. In these traditional PVDs, SPR-induced light concentration generally occurs at the interface between the metallic nanostructure and dielectric layer. A fundamental understanding of plasmonic processes at the metal/dielectric interface remains unclear, largely because such interfaces are not well defined in most PVDs. This is especially so for DSSCs, where metal nanostructures and mesoporous TiO_2_ are randomly mixed^23^,^24^. In the current study, we designed a PVD, consisting of a layer of metal nanoparticles (NPs) assembled on an atomically flat TiO_2_ dielectric/dye/graphene interface (Fig. 1a). Each interface was precisely controlled at the atomic level. This system allows the mechanism of interface-engineered plasmonic enhancement of the photovoltaic conversion to be studied.

## Results and Discussion

### Finite-difference time-domain (FDTD) simulations

**Figure 1:**
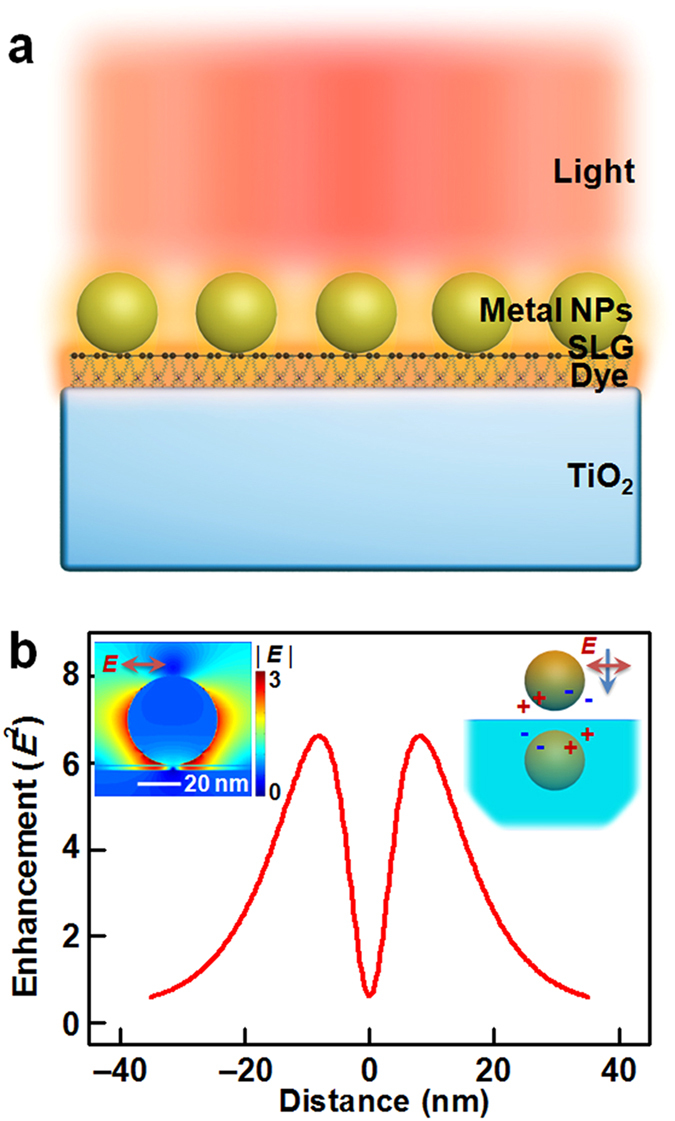
Substrate-induced interfacial plasmonics. (**a**) Schematic representation of substrate-induced interfacial plasmonics for photoexciting a layer of dyes between a TiO_2_ dielectric substrate and single-layer graphene (SLG). (**b**) Optical intensities at the TiO_2_ surface in the TiO_2_/SLG/NP system, calculated by two-dimensional FDTD simulations. The left inset shows the side view of the electrical field distribution surrounding individual NPs in the TiO_2_/SLG/NP system. The right inset illustrates the mechanism of the substrate-induced image charge effect.

FDTD simulations were used to predict the spatial distribution of the electromagnetic field in the PVD. The simulated model consisted of periodic spherical plasmonic NPs of radius 20 nm^26^, a single-layer graphene (SLG)^27^, and an infinite TiO_2_ dielectric substrate (*ε*_s_ = 7.34)^28^,^29^. The TiO_2_ substrate was separated from the SLG layer by a gap of 2 nm. The global electromagnetic field distribution at the TiO_2_/SLG/NP interface under parallel polarized light excitation is shown in Fig. 1b (left inset). Compared with the system without the TiO_2_ dielectric (Supplementary Fig. S1), the adjacent TiO_2_ dielectric significantly deflected the local electromagnetic field surrounding the particles to the TiO_2_/SLG interface. As shown in the optical intensity spectrum along the TiO_2_/SLG interface (Fig. 1b), a maximum electromagnetic field energy enhancement of ~6.6 times was achieved. This is much higher than that in the control system without the TiO_2_ dielectric (~2.0 times, Supplementary Fig. S1). The reason for this substrate-induced interfacial plasmonic enhancement is qualitatively explained by the image charge picture in Fig. 1b, right inset^30^,^31^. The adjacent dielectric substrate screened the electromagnetic field of the plasmonic particles. This is equivalent to the potential generated from its image charges in the substrate. As the plasmon of particles couples with its image charges, the electromagnetic field at the interface is enhanced. The magnitude of the image charges depends on the permittivity of the substrate with a factor of (*ε*_s_ −1)/(*ε*_s_ + 1). The large permittivity of TiO_2_ (*ε*_s_ = 7.34)^28^,^29^ dielectric led to a strong image charge effect and large plasmonic hybridization. This concentrated the electromagnetic field at the TiO_2_/NP interface.

Another reason for the substrate-induced interfacial plasmonics was the transparency of the SLG between the TiO_2_ and NPs. SLG is a monolayer-thick Dirac material, possessing both dielectric and semi-metallic properties^32^,^33^. It has high field transparency and also little image charge effect. The high field transparency meant that SLG did not shield the electromagnetic field at the TiO_2_/graphene interface, and thus the image charge effect of the TiO_2_ substrate. This field transparency deflected and concentrated the SPR effect at the dye layer between the TiO_2_ and SLG (Fig. 1b, left inset). Calculation without SLG (Supplementary Fig. S2) indicated that graphene enhanced plasmon hybridization at the interface. This was likely due to the involvement of intrinsic graphene plasmonics^34^ and/or the NP/SLG interfacial potential. This property of graphene, combined with its high electrical conductivity and light transparency^27^,^35^, was then exploited in a transparent photo-cathode^36^. The cathode was used to collect holes photo-generated from excited dyes, without interfering with the interfacial plasmonic enhancement of the NPs.

### Fabrication and characterizations of PVDs

**Figure 2:**
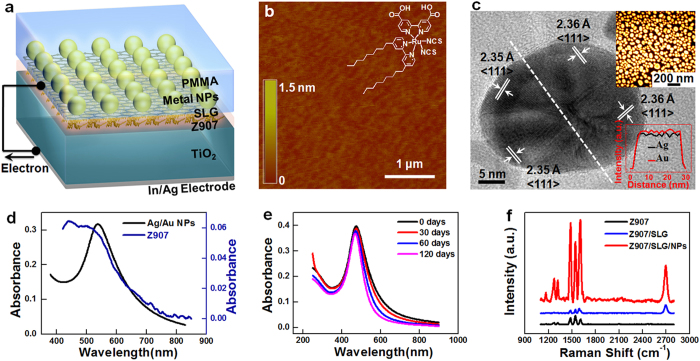
Fabrication and characterizations of PVDs. (**a**) Schematic illustration of the device structure. (**b**) AFM image of the rutile TiO_2_ (001) surface showing a root mean square (RMS) roughness of 0.81 Å. The inset shows the chemical structure of Z907. **(c)** HRTEM image of Ag/Au alloy nanoparticles. The upper inset is a SEM image of a Ag/Au NP film, obtained by annealing an 8-nm-thick Ag/Au alloy film (Ag:Au = 1:1). The lower inset is the elementary composition analysis along the white dashed line, which was calculated from EDX spectroscopy. (**d**) Absorption spectra of an Ag/Au (1:1) NP film on PMMA-supported graphene (black line) and a Z907 dye layer assembled on a TiO_2_ substrate (blue line). (**e**) Time-dependent absorption spectra of an Ag/Au (1:1) NP film on a quartz substrate, upon exposure to air at room temperature. (**f**) Raman spectra of Z907 on TiO_2_, Z907 on SLG (Z907/SLG), and Z907 on SLG/(Ag/Au) NPs (Z907/SLG/NPs).

To illustrate the substrate-induced interfacial plasmonic enhancement, we fabricated a PVD containing a TiO_2_/Dye/SLG/NP heterostructure. A schematic illustration of its structure is shown in Fig. 2a, and its fabrication is detailed in the Supplementary Information. In brief, atomically flat single-crystalline rutile TiO_2_ (001) (Fig. 2b) was used as the electron separating/transporting layer. Then 100-nm-thick In and 100-nm-thick Ag were thermally evaporated onto the back side of the TiO_2_ crystals, to form back Ohmic contacts. These contacts were connected to an external circuit using copper foils. Z907 is a typical amphiphilic ruthenium sensitizer (Fig. 2b inset) widely used in DSSCs^37^. Z907 was assembled on the TiO_2_ surface by dipping the substrate in a Z907 solution. High-quality SLG was grown on a copper foil by low-pressure chemical vapor deposition under optimized conditions^38^. A layer of Ag/Au alloy NPs was then formed on the graphene, by annealing 8-nm-thick Ag/Au alloy films that were thermally evaporated on the SLG surface with an Ag/Au ratio of 1:1. Scanning electron microscopy (SEM) images (Fig. 2c upper inset and Supplementary Fig. S3) indicated that the Ag/Au NP film had good uniformity, with an average NP diameter of ~38.1 nm and particle density of ~300 /μm^2^. High-resolution transmission electron microscopy (HRTEM) and energy-dispersive X-ray spectroscopy (EDX) characterization of individual NPs indicated that Ag and Au atoms were homogeneously distributed throughout the polycrystalline NPs. The top of the Ag/Au NPs was a layer of poly(methyl methacrylate) (PMMA). PMMA acted as a supporting layer during SLG/NP transfer, and a protective layer for photovoltaic measurements.

The black curve in Fig. 2d is the absorption spectrum of the Ag/Au NPs in a PMMA matrix. The localized SPR peak is apparent at ~536 nm. This value matched the absorption peak of Z907 on the TiO_2_ surface (Fig. 2d, blue), which was important for maximizing the light utilization and therefore efficiency. Using an Ag/Au alloy allowed Au atoms to protect the Ag NPs from oxidation^39^. The Ag/Au NPs remained stable following long exposure to air (Fig. 2e and Supplementary Fig. S6), which was necessary for stable SPR enhancement. The formation of Ag/Au NPs on the graphene surface resulted in negligible damage to the graphene layer. The Raman spectrum of SLG/NP indicated only a minor D peak at ~1356 cm^−1^ (Supplementary Fig. S7)^40^. The peaks of Z907 at 1200–1650 cm^–1^ in the Raman spectra of Z907/SLG/NP samples were significantly enhanced (Fig. 2f), compared with the spectra of Z907 on TiO_2_ and Z907 on SLG. This indicated that the Ag/Au NPs converged light to the Z907 layer below the graphene^41^,^42^. In summary, the above analyses demonstrated a precisely controlled TiO_2_/Dye/SLG/NP heterostructure, suitable for studying the interfacial plasmonic enhancement of photovoltaic conversion.

### Photovoltaic performances

**Figure 3:**
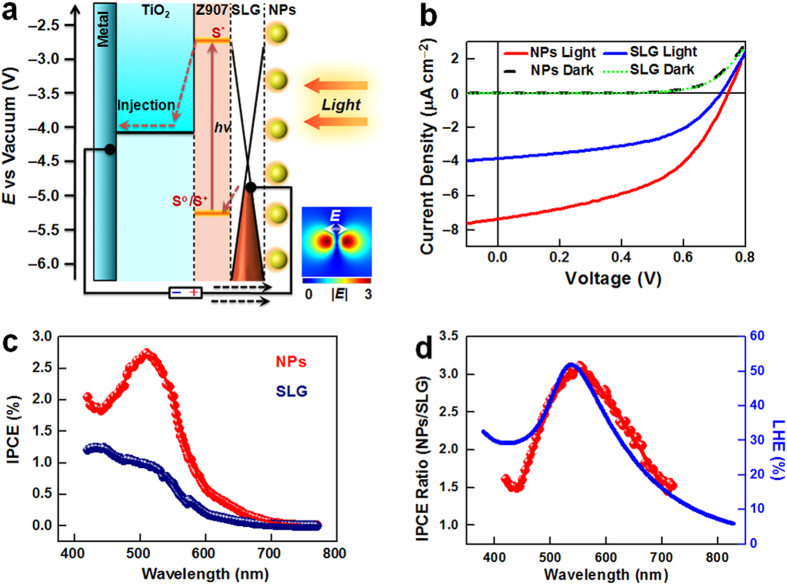
Photovoltaic performances. (**a**) Schematic illustration of the mechanism of substrate-induced interfacial plasmonic enhancement. The inset is the top view of the simulated electrical field distribution at the TiO_2_/SLG/NP interface. (**b**) Current-voltage characteristics of a PVD with a TiO_2_/Z907/SLG/NP interface (NPs), and a control device with a TiO_2_/Z907/SLG interface (SLG), in the dark and under 100 mW cm^−2^ broadband irradiation (>420 nm). **(c)** IPCE spectra of the working (red) and control (blue) devices in Fig. 3b. (**d**) Comparison of the IPCE(NPs)/IPCE(SLG) ratio-wavelength characteristics (red) with the light-harvesting efficiency (LHE) spectrum (blue) of an Ag/Au NP film.

The mechanism of the TiO_2_/Z907/SLG/NP device is shown in Fig. 3a. Z907 dye absorbed light to generate excited electrons, which were injected into the TiO_2_ layer. Injected electrons transported to the electron-collecting back electrode, and then into the external circuit. Finally, photo-excited Z907 was regenerated by electron feedback from the graphene layer, thus completing the photoelectric conversion. During charge separation/transport, plasmonic NPs on the surface of the SLG concentrated photon energy at the light-absorbing interface, thus enhancing the photo-excitation of Z907. This was shown by the simulated electrical field distribution at the TiO_2_/SLG/NP interface in Fig. 3a inset. Figure 3b shows the current-voltage (*I*–*V*) characteristics of a typical PVD containing Ag/Au NPs, and a control device without Ag/Au NPs, in the dark and under broadband visible (>420 nm) irradiation (100 mW cm^–2^). In the dark, the *I*–*V* curves of both devices showed similar rectifying characteristics, indicating that a built-in electric field formed at the TiO_2_/dye/SLG interface. This was necessary to effectively separate photo-generated electrons and holes from Z907, for photovoltaic conversion. The working device had a short-circuit current density (*J*_SC_) of ~7.36 μA cm^–2^ under visible light irradiation, which was ~1.9 times larger than that of the control device (~3.83 μA cm^–2^). This was attributed to SPR enhancement of Ag/Au NPs, as discussed above. The open-circuit photovoltage (*V*_OC_) of the working device (~0.743 V) was larger than that of the control device (~0.715 V), probably due to the p-doping effect of graphene by the Ag/Au NPs^43^.

Incident photon-to-current conversion efficiencies (IPCE) of the devices were measured as a function of wavelength to investigate the origin of the enhanced photoelectric conversion. IPCE values were calculated from *J*_SC_ values under the corresponding monochromatic irradiation. The IPCE spectrum of the control device (Fig. 3c, blue) at 420–700 nm exhibited a similar profile to the absorption spectrum of the Z907 film (Fig. 2d, blue). This indicated that the photo-excitation of Z907 significantly contributed to the device’s photocurrent. The IPCE of the working device (Fig. 3c, red) showed a significant enhancement at 420–700 nm, compared with the control device. This was clear evidence of the interfacial plasmonic enhancement effect. Ratios of IPCE values of the working and control devices (IPCE(NPs)/IPCE(SLG)) were calculated at corresponding wavelengths (Fig. 3d, red). A maximum enhancement of 3.1 times was observed at the 540-nm peak position. The spectral shape and peak position of the IPCE ratio-wavelength characteristics matched those of the light-harvesting efficiency (LHE) spectrum of the Ag/Au NP film (Fig. 3d). This suggested that it was the SPR of the NPs that concentrated the optical energy at the interface, enhancing the photo-excitation of the dyes and improving the photoelectric conversion efficiency.

### Nanoparticle component dependence

**Figure 4:**
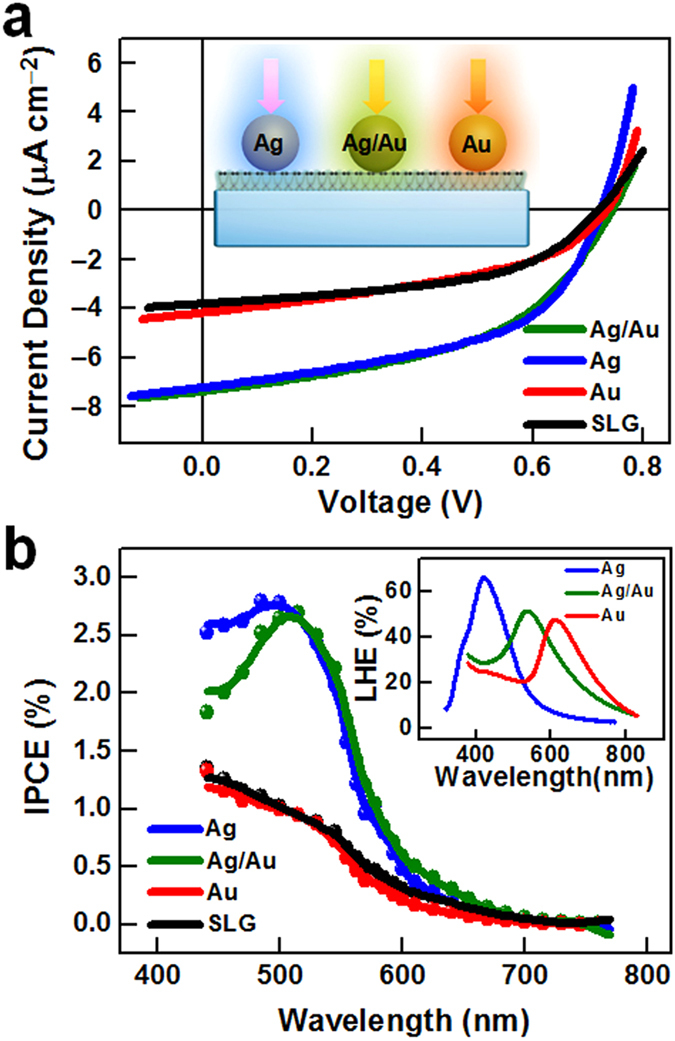
Component-dependent SPR enhancement. (**a**) Current-voltage characteristics of PVDs containing Au (red), Ag (blue) and Ag/Au (1:1) alloy NPs (olive), and the control device without metal NPs (SLG: black), under 100 mW cm^−2^ broadband visible (>420 nm) irradiation. The inset shows the device structure with different metal NPs. (**b**) Corresponding IPCE spectra of the devices in Fig. 4(a). The inset shows the light-harvesting efficiency (LHE) spectra of Au, Ag, and Ag/Au (1:1) alloy NP films.

The localized SPR absorption peaks of the NPs were then tuned by changing the Ag/Au ratio. The localized SPR peaks of Ag (Supplementary Fig. S8), Ag/Au (1:1) and Au (Supplementary Fig. S9) in PMMA matrices (Fig. 4b inset) exhibited red-shifts from 423 to 615 nm, with increasing Au content. Figure 4 shows the *I*–*V* curves and corresponding IPCE spectra of PVDs containing different NPs (Fig. 4b). The IPCE of the device containing Ag NPs exhibited a similar enhancement to that containing Ag/Au NPs, but the IPCE peak of the former was blue-shifted. This was consistent with the changes in the SPR absorption spectra of Ag and Ag/Au NPs. Negligible IPCE enhancement was exhibited by the device containing Au NPs. This was because the localized SPR spectrum of the Au NPs did not well match the absorption spectrum of the Z907 film. These results supported the earlier conclusion that the PVD efficiency enhancement was due to the interfacial plasmonic enhancement of Ag or Ag/Au alloy NPs, rather than doping and/or intrinsic plasmon-induced hot-electron effects of the NPs^44^. The Ag/Au (1:1) NP film was far more stable than the Ag NP film, as demonstrated in Fig. 2e and Supplementary Fig. S6.

### Nanoparticle size dependence

**Figure 5:**
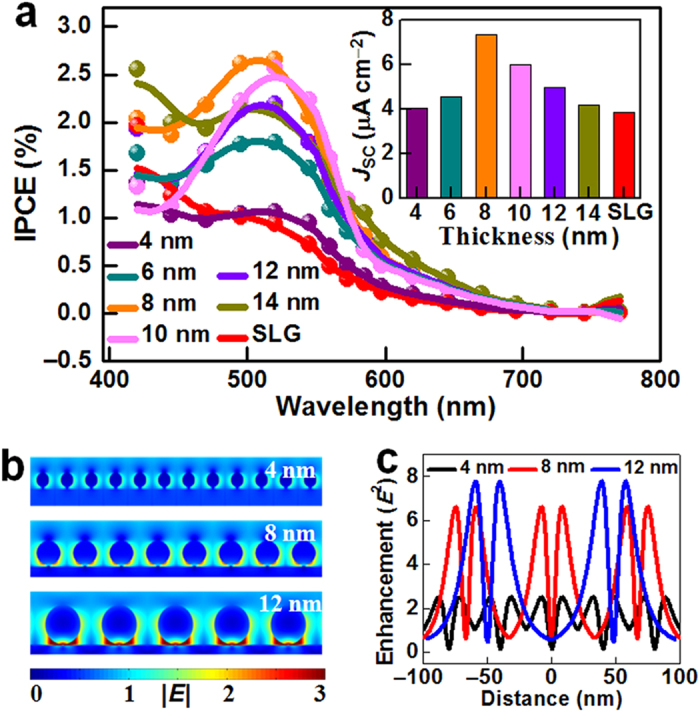
Size-/spacing-dependent interfacial plasmon enhancement. (**a**) IPCE spectra of PVDs containing different metal NPs. PVDs were formed by annealing Ag/Au (1:1) alloy films of thicknesses of 0 (SLG), 4, 6, 8, 10, 12 and 14 nm. The inset is the short-circuit currents of the corresponding devices, measured under 100 mW cm^–2^ visible light irradiation. (**b**) Side views of electrical field distributions of different TiO_2_/SLG/NP systems (top: 4-nm-thick NPs with 21-nm diameter and 40-nm spacing; middle: 8-nm-thick NPs with 38-nm diameter and 67-nm spacing; bottom: 12-nm-thick NPs with 64-nm diameter and 100-nm spacing). The field images were obtained from two-dimensional FDTD calculations. (**c**) Corresponding light intensities as a function of distance at the interfaces of TiO_2_/SLG/NP systems with different NP thicknesses.

The size of metal NPs and the plasmon resonance coupling between them reportedly greatly influences the SPR characteristics of the NPs, and the field distribution surrounding them^10^,^16^,^17^. To investigate the effects of these two parameters (i.e. NP size and interparticle coupling) on the current substrate-induced interfacial plasmonic enhancement, Ag/Au (1:1) NPs with different particle sizes and interparticle spacings were fabricated, by annealing Ag/Au (1:1) films of different thicknesses. SEM (Supplementary Figs S10 and S11) indicated that as the Ag/Au film thickness increased from 4 to 14 nm, the average particle diameter increased from 21 to 133 nm, and the particle density decreased from 740 to 24 /μm^2^. The latter represented an increased interparticle spacing. The localized SPR absorption peaks of Ag/Au NPs in the PMMA matrix broadened and became more intense with increasing film thickness (Supplementary Fig. S12). A red-shift in the peak maximum from 521 to 573 nm was also observed. These observations were due to the effect of the NP size on SPR^45^. Figure 5a shows the IPCE performance of the devices containing different metal NPs. The IPCE peak values gradually increased and then declined with increasing NP diameter, with a maximum IPCE observed at a diameter of 8 nm. The Ag/Au (1:1) NPs were highly stable in air, which was another reason for 8-nm-thick Ag/Au NPs being selected for the plasmonic antenna in this study.

To explain the variations in IPCE peaks, field distributions of the TiO_2_/SLG/NP system were simulated by FDTD simulations, using the different metal NP parameters (Fig. 5b): 21-nm diameter with 40-nm interparticle center-to-center spacing for 4-nm-thick NPs (denoted 4-nm NPs); 38-nm diameter with 67-nm spacing for 8-nm-thick NPs; 64-nm diameter with 100-nm spacing for 12-nm-thick NPs. Without considering interparticle coupling, the energy intensity at the interface was greatly enhanced. The maximum intensity increased from ~1.8 times for 4-nm NPs, to ~7.4 times for 8-nm NPs, to ~21 times for 12-nm NPs (Supplementary Fig. S15). In general, the localized SPR absorption of the metal NPs is enhanced with increasing size. The substrate-induced plasmon coupling is weakened as the localized SPR of metal NPs shifts further from the substrate. This means that the SPR enhancement of the metal NPs dominates the overall plasmonic effect, in the absence of interparticle coupling. When interparticle coupling was introduced into the system, a further enhancement in interfacial energy intensity from 1.8 to 2.5 times was observed for 4-nm NPs (Fig. 5c and Supplementary Fig. S15). This was because interparticle plasmon resonance coupling between individual NPs with smaller diameters occurred more near to the TiO_2_ substrate, which further strengthened the interfacial plasmonic enhancement. Increasing the NP diameter shifted the interparticle coupling electromagnetic field further from the TiO_2_ substrate. Thus, the interparticle coupling had a lower contribution to the interfacial energy enhancement. Interparticle coupling also competed with substrate-induced plasmon coupling, which weakened the plasmonic enhancement at the TiO_2_/SLG interface. For instance, in the 12-nmNP system with 67-nm diameter and 100-nm spacing, the interparticle coupling was sufficiently strong to weaken the interfacial energy enhancement from ~21 to ~7.9 times (Fig. 5c and Supplementary Fig. S15). Therefore, both the size and interparticle spacing of metal NPs influenced the optical energy concentration at the interface by balancing the competition between localized SPR, substrate-induced plasmon coupling, and interparticle coupling. This accounted for the fact that TiO_2_/Z907/SLG/NP device containing 8-nm-thick NPs exhibited the maximum plasmonic enhancement. These results provide reliable insights into maximizing the performance of PVDs, by designing precise interfaces with optimal NP sizes and spacings.

## Conclusion

In conclusion, a PVD containing a TiO_2_/Dye/SLG/NP interface was developed, to aid fundamental understanding of the effect of SPR enhancement on photovoltaic conversion. Theoretical and experimental results indicated that using transparent graphene as a photocathode gave significant substrate-induced plasmonic coupling between plasmonic NPs and TiO_2_. Thus, the optical energy was concentrated at the TiO_2_/NP interface, which enhanced the photo-excitation of the dyes and thus the photoelectric conversion. Balancing competition between substrate-induced coupling and interparticle coupling was demonstrated, by optimizing particle sizes and spacing to maximize substrate-induced plasmonic enhancement. This physical understanding of interfacial plasmonic enhancement offers new approaches and mechanistic insights for designing efficient solar cells using plasmonic nanostructures with well-defined interfaces. The proved capability to manage and concentrate light will also promote the development of ultrasensitive plasmon-based sensors and photocatalysts.

## Methods

### Details of Device Fabrications

#### Graphene Growth

High-quality single layer graphene (SLG) was grown by a low-pressure chemical vapor deposition (LPCVD) method with methane as the carbon-containing precursor under optimal conditions^38^. In detail, Cu foils, which were pretreated by acetic acid, were put into a split tube furnace for graphene growth. During graphene growth, copper foils were firstly annealed at the temperature of 1045 °C under 10 sccm H_2_ flow 60 min; then, gas atmosphere with 1 sscm CH_4_ and 2 sccm H_2_ (95 mTorr) was used for graphene growth for 15 min; after that, the temperature was quickly dropped to room temperature with 10 sccm H_2_ protection.

#### Metal Nanoparticle Preparation

Ag/Au alloy thin films with proper Ag/Au volume ratios and thicknesses were first uniformly thermally evaporated onto the surface of graphene on copper foil substrates at a rate of 0.1 Å/s under a pressure of 2 × 10^−4^ Pa. After that, the samples were annealed at a temperature of 300 °C for 30 min with H_2_ (600 sccm) and Ar (600 sccm) as the protective gas atmosphere, which lead to the formation of a uniform Ag/Au nanoparticle (NP) layer on the surface of graphene.

#### Back Electrode/TiO_2_/Z907 Layer Preparation

One-side mechanically-polished rutile TiO_2_ (001) single crystals with atomically flat surface were obtained from MTI Corporation. 100 nm of In and 100 nm of Ag were in turn thermally evaporated onto the rough back side of the TiO_2_ crystals, which were used as Ohmic contact back electrodes. Before dye assembly, the surface of the TiO_2_ layer was pretreated by aqueous HF solution (30% HF, 1 min) for chemical polishing and was further etched with oxygen plasma (15 Pa, 30 W; 1 min) for surface hydroxy activation. After that, Z907 dye assembly on the surface of TiO_2_ was realized by immersing the samples into a 0.3 mmol/L solution of Z907 in acetonitrile and t-butanol for 24 hours and further rinsing with copious amount of acetonitrile. Finally, the In/Ag side of the samples were mounted on a copper foil wire, which was fixed on a glass sheet with conductive tap, with Ga/In eutectic; and the back electrode was further sealed with epoxy (Epotek 377) and dried for one hour to solidify.

#### SLG/NP Layer Formation

For SLG/NP layer assembly, a layer of poly(methyl methacrylate) (PMMA) (MicroChem 950 PMMA A6), which was used as a protective and support layer, was spin-cast on the surface of prepared Cu/SLG/NP samples at 4000 rpm for 30 seconds and baked at 180 °C for 3 min. As both sides of Cu foils were growth with graphene, the back side of the Cu/SLG/NP/PMMA samples was further etched by oxygen plasma to remove the residual graphene. Then, the copper layer in the samples was wet-etched by 1 M (NH_4_)_2_S_2_O_8_ aqueous solution for about 8 hours, producing the SLG/NP/PMMA film floating in the etchant. After that, the floating film was in turn washed with copious ultrapure water and isopropanol. Finally, the SLG/NP/PMMA film was transferred on the surface of TiO_2_/Z907 layers and baked at 80 °C for 5 min for close contact. In addition, the PMMA-supported graphene layer was further connected to the external circuit with copper foils.

### Details of device characterizations and measurements

#### Morphology characterizations

The morphology of metal nanoparticle films was studied by a field-emission scanning electron microscope (SEM; Hitachi Limited, S4800). The crystal structure and elemental composition of metal nanoparticles was analyzed with a high-resolution transmission electron microscope (HRTEM; FEI, Tecnai F30). Atomic force microscopy (AFM) measurements were carried out with a tapping mode AFM (Digital Instruments NanoscopeIII a controller and a multimode SPM).

#### Optical characterizations

The light-absorption properties of the samples were measured by a Lambda 35 ultraviolet–visible (UV-vis) spectrometer equipped with an integrating sphere (Lambda 950, Perkin Elmer). Raman measurements were carried out by using a micro-Raman spectroscope (Renishaw 1000), with an excitation wavelength of 632.8 nm.

#### Photoelectrical measurements

The current-voltage characteristics of the photovoltaic devices were measured by using a semiconductor characterization system (Agilent 4155C) in the dark and under AM 1.5 simulated solar light irradiation (Science Tech) through a UV light filter (420 nm cut-off wavelength), whose light intensity was adjusted to 100 mW/cm^2^.

For the incident photon-to-current efficiency (IPCE) spectroscopic measurements, a computer-controlled grating monochromator (Zolix Omni-λ150) with a 150 W Xe lamp was used for providing the monochromatic light illumination to the samples through an optical filter. The light power and corresponding wavelengths for the illuminated monochromatic light was measured with an OPT-2000 spectrophotometer. The corresponding photocurrent was measured with the Agilent 4155C semiconductor parameter analyzer at current time mode. The IPCE spectra were calculated from the measured photocurrents, corresponding monochromatic light wavelength (λ) and light power with the following formula:


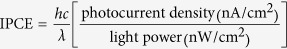


where *h* is the Planck’s constant and *c* is the speed of light. All the photoelectrical measurements were performed on at least three samples and each sample was tested at least three times. The light intensity-dependent performance of the optimized model photovoltaic devices was shown in the supplementary information (Supplementary Fig. S16).

### Theoretical Calculations

Finite-difference time-domain (FDTD) simulations were carried out by using a commercially available software package^46^, lumerical FDTD solutions. The simulation cell consists of a TiO_2_ substrate, a single layer graphene on the top, and Ag/Au nanoparticles, which arranged in a square lattice with a lattice constant *P*. The cell was normally illuminated by a broadband plane wave source on the above. This simulation was carried out upon a single unit cell of the nanoparticle array, and periodic boundary conditions were applied on the horizontal directions, and PML boundary conditions on the vertical directions. For the calculations, we take the experimentally measured optical constants of Ag from ref. 47 to simulate the Ag/Au material, and use a constant refractive index of 2.71 for TiO_2_^29^. The local field intensity of the systems was monitored at the vertical cross section of a unit cell parallel to the polarization direction of the incident light, and normalized to an identical reference system without the nanoparticles.

## Additional Information

**How to cite this article**: Li, X. *et al.* Substrate-induced interfacial plasmonics for photovoltaic conversion. *Sci. Rep.* **5**, 14497; doi: 10.1038/srep14497 (2015).

## Supplementary Material

Supplementary Information
