# Supplementary material for: Substrate-induced interfacial plasmonics for photovoltaic conversion
Source: Sci Rep. 2015 Sep 28;5:14497. doi: 10.1038/srep14497 (PMC4585970; doi:10.1038/srep14497)
Supplement: Supplementary Information [file srep14497-s1.pdf]

Supplementary Information for

Substrate-induced interfacial plasmonics for photovoltaic conversion

Xinxi Li[1†], Chuancheng Jia[2†], Bangjun Ma[2], Wei Wang[3], Zheyu Fang[3], Guoqing Zhang[1]* and Xuefeng Guo[2,4]*

[1]School of Materials and Energy, Guangdong University of Technology, Guangzhou 510006, P. R. China.

[2]Center for Nanochemistry, Beijing National Laboratory for Molecular Sciences, State Key Laboratory for Structural Chemistry of Unstable and Stable Species, College of Chemistry and Molecular Engineering, Peking University, Beijing 100871, P. R. China.

[3]State Key Lab for Mesoscopic Physics, School of Physics, Peking University, Beijing 100871, P. R. China.

[4]Department of Materials Science and Engineering, College of Engineering, Peking University, Beijing 100871, P. R. China.

*Correspondence and requests for materials should be addressed to X.G. (guoxf@pku.edu.cn) or G.Z. (pdzgq008@126.com).

[†]These authors contributed equally to this work.

Supplementary Index

Figure S1. Theoretical simulation of the optical intensities below a single nanoparticle without TiO_2 substrates.

Figure S2. Effect of graphene on the interfacial energy enhancement.

Figure S3. SEM characterization of Ag/Au alloy nanoparticles.

Figure S4. Two-dimensional fast Fourier transform (FFT) pattern from the HRTEM image of Ag/Au nanoparticle (Figure 2c).

Figure S5. EDX analysis of Ag/Au nanoparticles.

Figure S6. Air stability of Ag and Ag/Au alloy nanoparticles.

Figure S7. Raman spectra of pure graphene and the graphene assembled with Ag/Au nanoparticles.

Figure S8. SEM characterization of Ag nanoparticles.

Figure S9. SEM characterization of Au nanoparticles.

Figure S10. SEM characterizations of Ag/Au nanoparticles with different sizes.

Figure S11. Statistic particle parameters of Ag/Au nanoparticles with different thicknesses.

Figure S12. Optical properties of Ag/Au nanoparticles with different sizes.

Figure S13. Current-voltage characteristics of the photovoltaic devices with different thicknesses of Ag/Au nanoparticles.

Figure S14. IPCE peak values for the photovoltaic devices used in Figure 5a.

Figure S15. Interparticle coupling effects on the interfacial plasmon resonance enhancement.

Figure S16. The light intensity-dependent performance of the optimized model photovoltaic devices with a single layer of Z907 and 12-nm-thick Ag/Au (1:1) NPs.

Table S1. Device characteristics for the photovoltaic devices assembled with different nanoparticles.

Table S2. Device characteristics for the photovoltaic devices assembled with Ag/Au nanoparticles of different thicknesses.

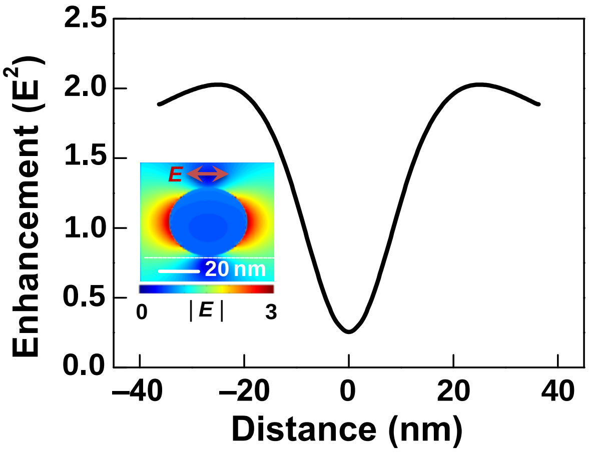

Figure S1. Theoretical simulation of the optical intensities below a single nanoparticle without TiO_2 substrates. The inset is the side view of the electrical field distribution surrounding individual NPs achieved by two-dimensional finite-difference time-domain (FDTD) calculation. Compared with the system with TiO_2 substrates (Figure 1b), the lower and symmetric energy intensity for the individual nanoparticle system was obtained, indicating that substrate-induced plasmonic hybridization plays the key role in the interfacial energy enhancement for the system with TiO_2 substrates.

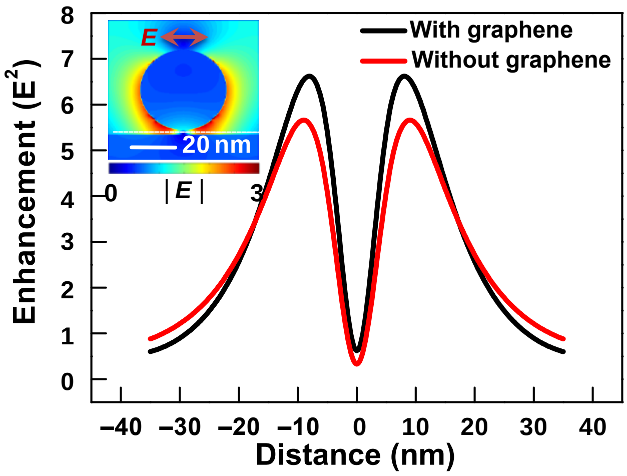

Figure S2. Effect of graphene on the interfacial energy enhancement. The curves show the interfacial energy intensity enhancements at the TiO_2 surface for the systems with and without graphene, respectively. The left inset is the side view of the electrical field distribution of metal nanoparticle/TiO_2 system, which has the same parameters as the nanoparticle/TiO_2 system with graphene. The systems were calculated by two-dimensional FDTD methods. By comparing the interfacial energy intensities of the systems with and without graphene, it can be observed that the presence of graphene not only has little effect on the energy intensity below graphene, which is due to the electromagnetic field transparency of the graphene at the visible light spectrum,[S1] but also slightly improved the interfacial energy intensity due to the involvement of intrinsic graphene plasmonics and/or the NP/SLG interfacial potential.

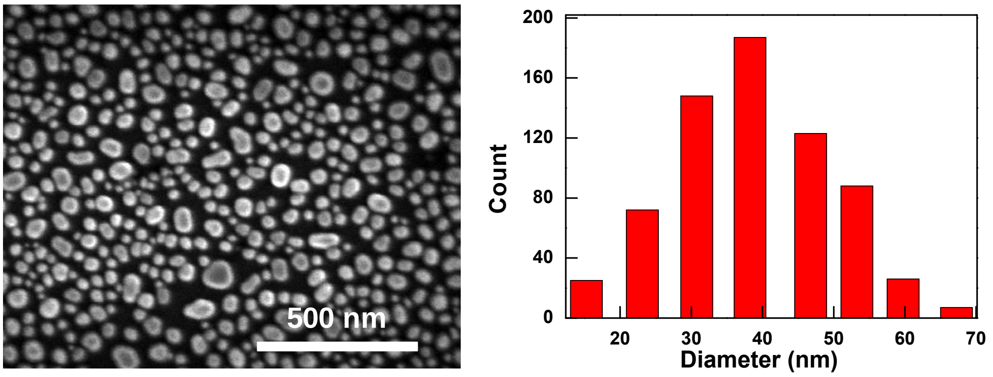

Figure S3. SEM characterization of Ag/Au alloy nanoparticles. The left is a SEM image of Ag/Au nanoparticles obtained by annealing 8-nm thick homogenous Ag/Au alloy thin films (Ag/Au = 1:1). The right is the size distribution histogram showing an average diameter of 38.1 nm and a particle density of 300/μm^2.

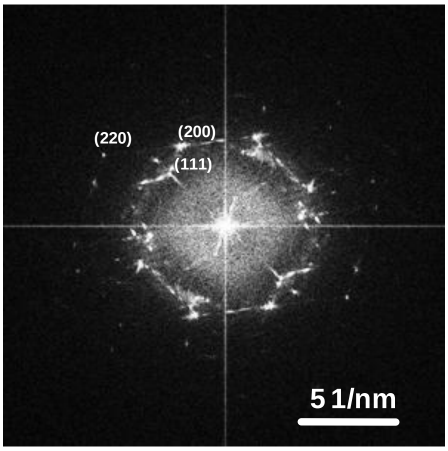

Figure S4. Two-dimensional fast Fourier transform (FFT) pattern from the HRTEM image of Ag/Au nanoparticle (Figure 2c). The FFT pattern can be indexed to the diffractions from (111), (200), and (220) planes of the nanoparticles, which indicates the uniform distribution of Ag and Au atoms in Ag/Au nanoparticles.

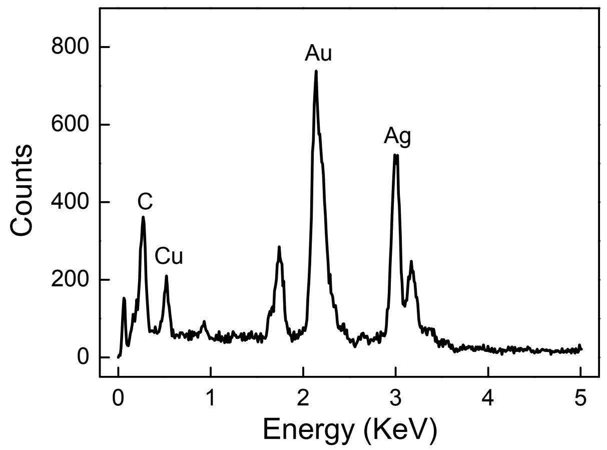

Figure S5. EDX analysis of Ag/Au nanoparticles. The energy dispersive X-ray (EDX) analysis of Ag/Au nanoparticles shows the presence of Ag and Au elements in the nanoparticles. By integrating the dispersive peaks for Ag and Au elements, it can be calculated that the molar ratio of Ag and Au elements is about 1:1.

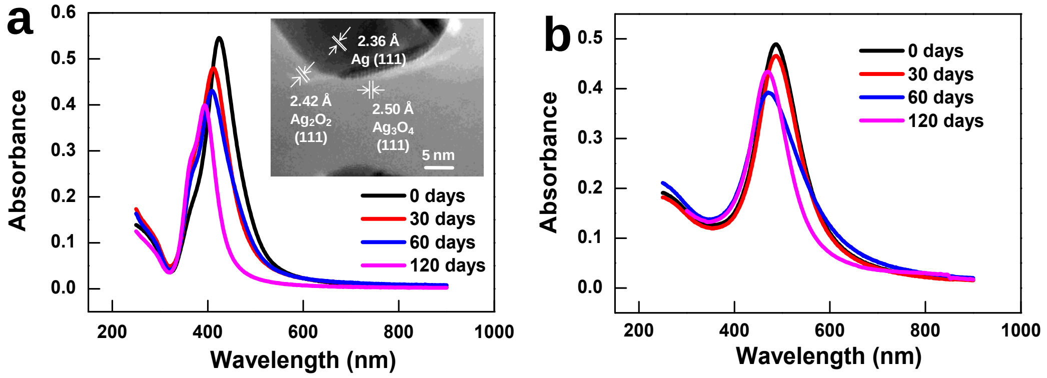

Figure S6. Air stability of Ag and Ag/Au alloy nanoparticles. These figures show the absorbance of pure Ag nanoparticles (a), which were obtained by annealing 8-nm thick Ag thin films, and Ag/Au alloy nanoparticles (b), which were obtained by annealing 8-nm thick Ag/Au alloy thin films (Ag/Au = 2:1), upon exposure to the air at room temperature for different times. For pure Ag nanoparticles, the localized SPR absorbance peak at about 420 nm showed a blue shift with the gradual peak intensity decrease, which should be ascribed to the oxidation of Ag in the air.[S2] Furthermore, from the HRTEM image of Ag nanoparticles after exposure to air for 120 days, the obvious surface oxidation of Ag nanoparticles was observed (Figure S6a inset). However, addition of Au atoms slowed down the Ag oxidation (Figure S6b, Ag/Au = 2:1). With the increase of Au ration up to 1:1 (Figure 2e), the existed Au atoms can effectively protect the oxidation of Ag so that Ag/Au alloy nanoparticles showed good air stability.

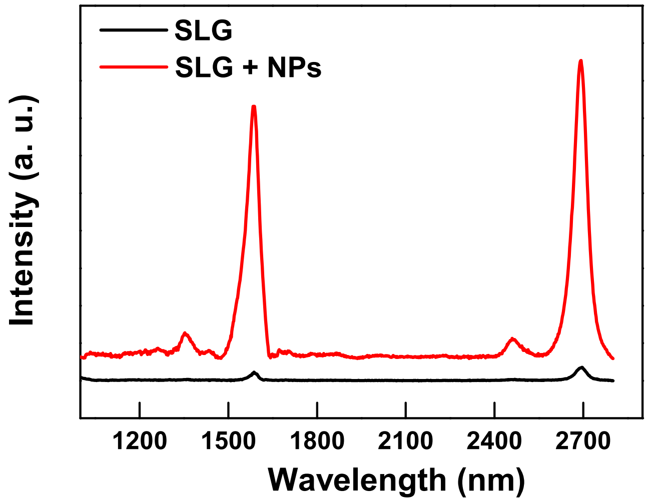

Figure S7. Raman spectra of pure graphene and the graphene assembled with Ag/Au nanoparticles. For the Raman spectrum of pure graphene (black line), narrow single symmetric 2D peak (~2694 cm^{-1}), small G/2D ratio, and negligible D peak indicate that the graphene is single-layered and of high quality.[S3] For the Raman spectrum of graphene assembled with Ag/Au nanoparticles, only a little D peak (~1353 cm^{-1}) can be observed, which indicates little destruction of graphene during Ag/Au nanoparticle formation. In addition, the Raman intensity of the graphene after nanoparticle assembly is much higher than that of pure graphene, which manifests that metal nanoparticles can enhance the energy intensity nearby the graphene layer.

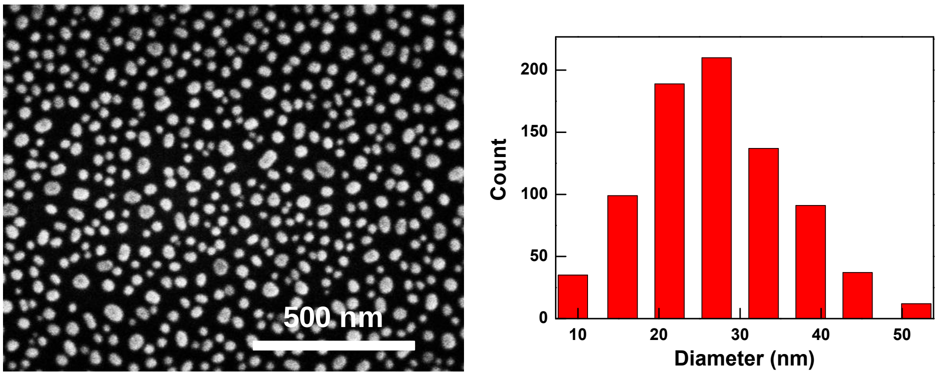

Figure S8. SEM characterization of Ag nanoparticles. The left is a SEM image of Ag nanoparticles obtained by annealing 8-nm thick pure Ag thin films. The right is the size distribution histogram showing an average diameter of 27.1 nm and a particle density of 399/μm^2.

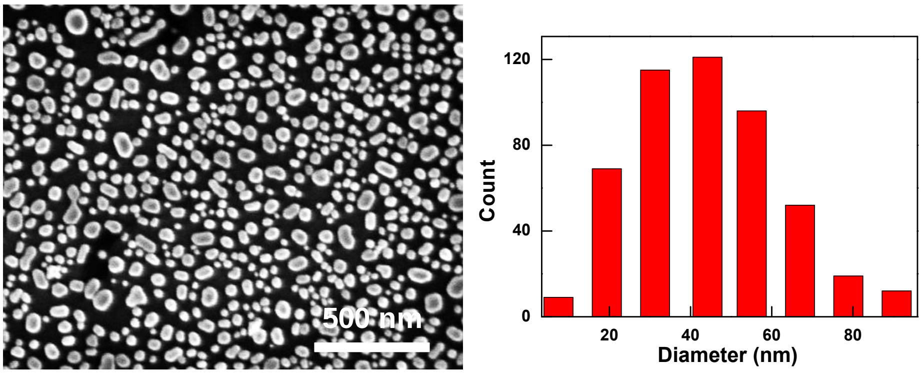

Figure S9. SEM characterization of Au nanoparticles. The left is a SEM image of Au nanoparticles obtained by annealing 8-nm thick pure Au thin films. The right is the size distribution histogram showing an average diameter of 43.9 nm and a particle density of 245/ μm^2.

Table S1. Device characteristics for the photovoltaic devices assembled with different nanoparticles.

	SLG	Au	Ag	Ag/Au
*J*sc (μA cm–2)	3.83	4.17	7.22	7.36
*V*oc (V)	0.717	0.738	0.721	0.743
FF	0.499	0.433	0.516	0.481

*J*sc is the short-circuit current; *V*oc is the open-circuit voltage; FF is the fill factor. SLG is the control photovoltaic device without metal nanoparticles; Au is the device with pure Au nanoparticles; Ag is the device with pure Ag nanoparticles; Ag/Au is the device with Ag/Au nanoparticles (Ag:Au = 1:1). The performances of photovoltaic devices were measured under 100 mW cm^{-2} visible light irradiation with a UV light filter (420 nm cut-off wavelength). Compared with the control device, the performance of the device with Au nanoparticles has little enhancement, which is because that the SPR absorbance peak of Au nanoparticles does not match the optical absorption spectrum of Z907. For Ag and Ag/Au systems, they have similar enhanced performances. However, due to the stability of Ag/Au nanoparticles, Ag/Au nanoparticles are more suitable as plasmonic antenna than Ag nanoparticles in our interfacial plasmon enhanced system.

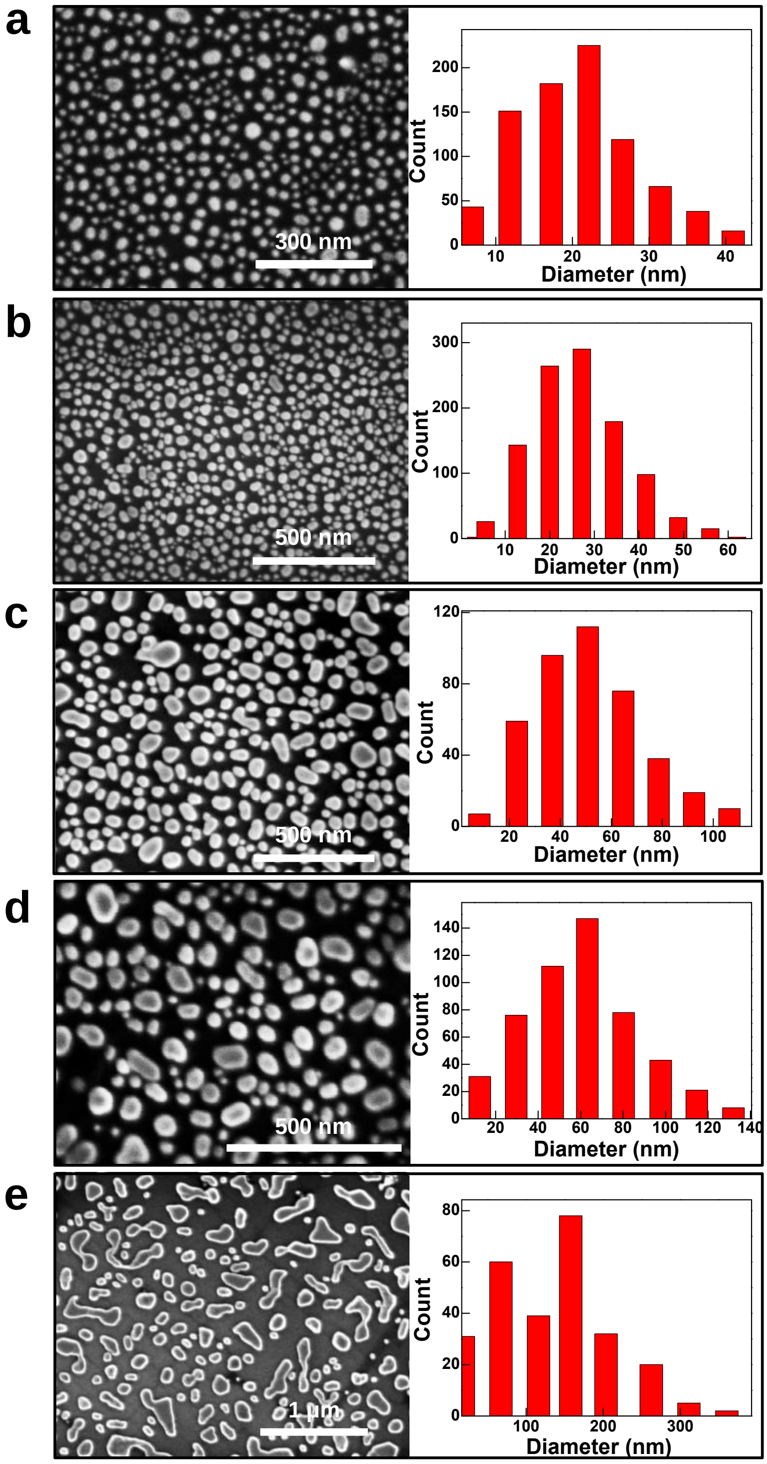

Figure S10. SEM characterizations of Ag/Au nanoparticles with different sizes. The left are the SEM images and the right are the size distribution histograms of Ag/Au nanoparticles obtained by annealing homogenous Ag/Au (1:1) alloy thin films with thickness of 4 nm (a), 6 nm (b), 10 nm (c), 12 nm (d) and 14 nm (e). The diameter and density parameters are summarized in Figure S11 below.

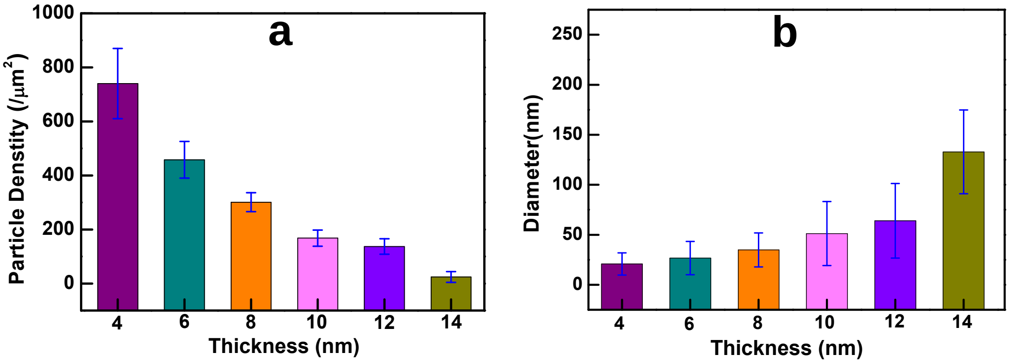

Figure S11. Statistic particle parameters of Ag/Au nanoparticles with different thicknesses. Figure a is the statistic average particle diameters and Figure b is the particle densities of Ag/Au nanoparticles, which were obtained by annealing homogenous Ag/Au (1:1) alloy thin films with different thicknesses. The error bar of average particle diameters is defined by the standard deviation over 3 trails. As shown in this figure, the particle densities are decreased with the increase of the original thickness of Ag/Au alloy thin films, which leads to the increased spacings between nanoparticles; in contrast, the average diameters of the nanoparticles increase with the increased original film thicknesses. Therefore, by modulating the original thickness of thin films before annealing, particle spacings and particle diameters of the nanoparticles can be effectively controlled.

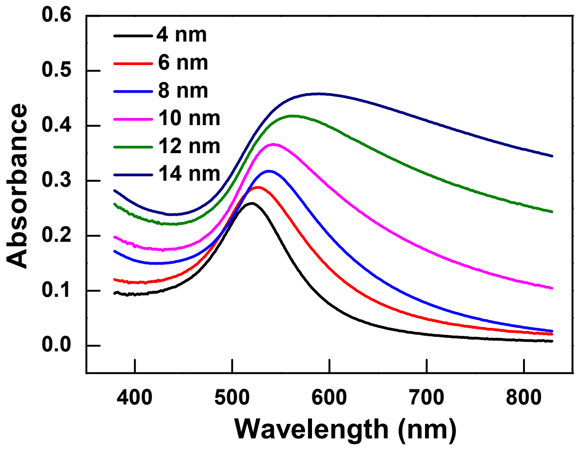

Figure S12. Optical properties of Ag/Au nanoparticles with different sizes. This figure shows the absorbance spectra of Ag/Au nanoparticles assembled on graphene with a PMMA protective layer, which were prepared by annealing homogenous Ag/Au (1:1) alloy thin films with different thicknesses. With the increase of the original thicknesses of Ag/Au alloy thin films, the SPR absorbance of Ag/Au nanoparticles increased and the position of the SPR absorbance peak had a small red shift from 521 nm to 573 nm, which also indicates the increased particle diameter of the nanoparticles.[S4]

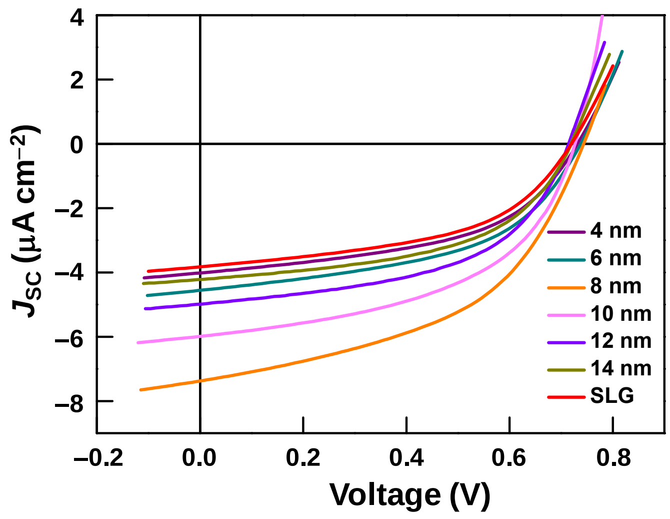

Figure S13. Current-voltage characteristics of the photovoltaic devices with different thicknesses of Ag/Au nanoparticles. This figure shows the current-voltage characteristics of the photovoltaic devices used in Figure 5a under 100 mW cm^{-2} broadband visible light (> 420 nm) illumination. We found that when the thickness increased, the photocurrents gradually increased and then declined with a maximal value at 8 nm thickness. The detailed parameters (J_{sc}, V_{oc}, and FF) of all the devices are summarized in Table S2 below.

Table S2. Device characteristics for the photovoltaic devices assembled with Ag/Au nanoparticles of different thicknesses.

	4 nm	6 nm	8 nm	10 nm	12 nm	14 nm	SLG
J_{sc} (μA cm^{-2})	4.02	4.54	7.36	5.99	4.97	4.19	3.83
V_{oc} (V)	0.728	0.733	0.741	0.729	0.721	0.718	0.719
FF	0.501	0.503	0.482	0.498	0.517	0.519	0.497

J_{sc} is the short-circuit current; V_{oc} is the open-circuit voltage; FF is the fill factor. "SLG" is the control photovoltaic device without metal nanoparticles. The original film thicknesses of Ag/Au nanoparticles varies from 4 nm to 14 nm.

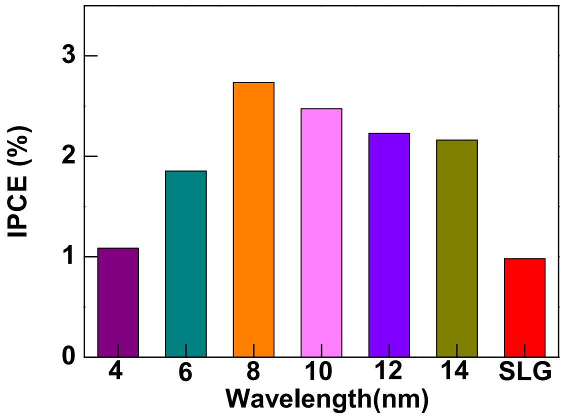

Figure S14. IPCE peak values for the photovoltaic devices used in Figure 5a.

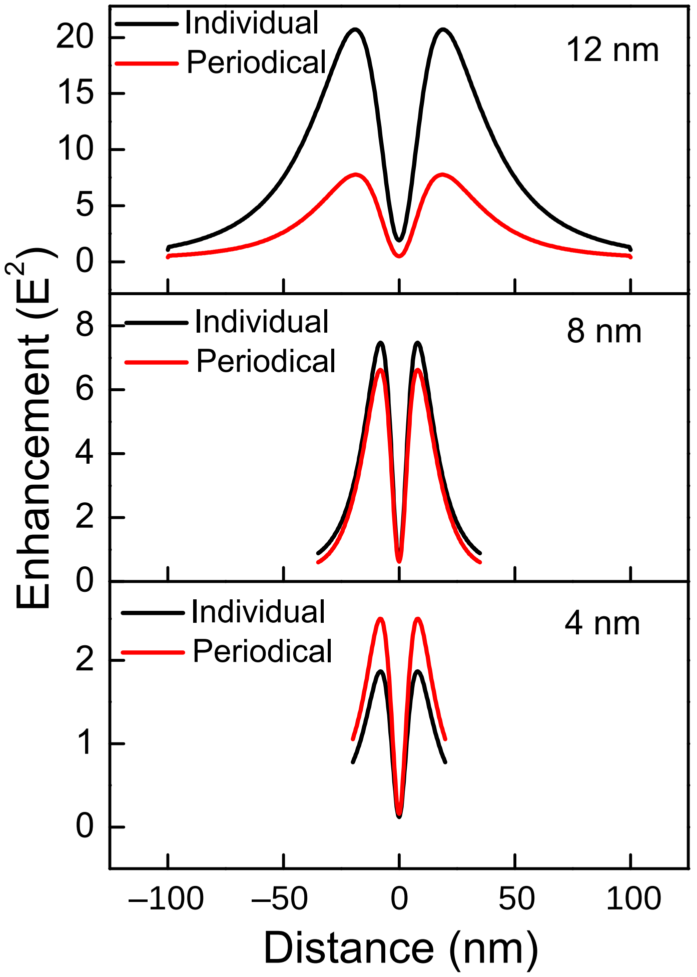

Figure S15. Interparticle coupling effects on the interfacial plasmon resonance enhancement. This figure shows the energy intensity distributions at the TiO_2/SLG/NP interface in different systems (bottom: 4-nm NPs with 21 nm diameter and 40 nm spacing; middle: 8-nm NPs with 38 nm diameter and 67 nm spacing; top: 12-nm NPs with 64 nm diameter and 100 nm spacing). The "Periodical" red lines represent the data obtained by considering interparticle coupling during the FDTD calculation; the "Individual" black lines represent the data without considering interparticle coupling during the FDTD calculation.

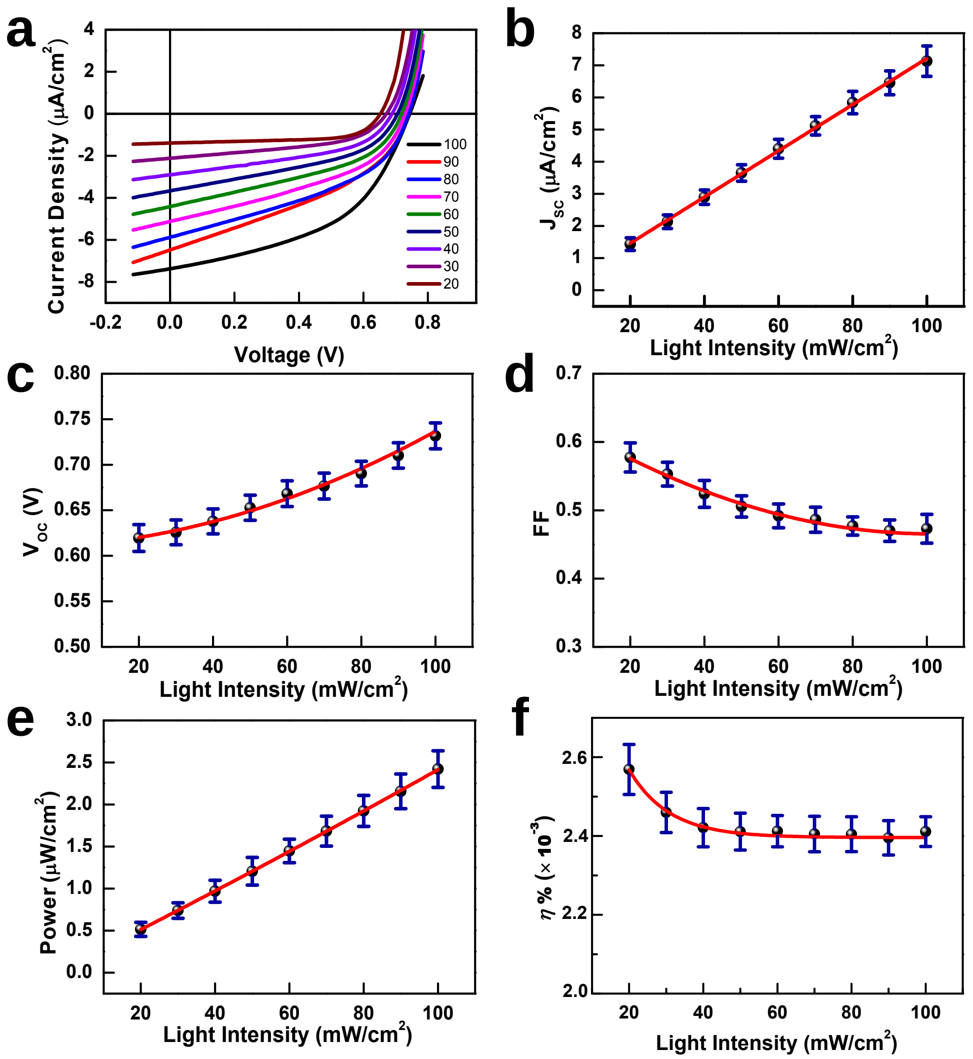

Figure S16. The light intensity-dependent performance of the optimized model photovoltaic devices with a single layer of Z907 and 12-nm-thick Ag/Au (1:1) NPs. **(a)** Typical original current-voltage characteristics of a photovoltaic device with the light intensity changing from 20 mV/cm^2 to 100 mV/cm^2. **(b)** Short-circuit currents (J_{sc}) of the photovoltaic devices as a function of the light intensity. It was found that J_{sc} changed linearly with the light intensity. **(c)** Open-circuit voltages (V_{oc}) of the photovoltaic devices as a function of the light intensity. The V_{oc} increased with the increase of the light intensity. **(d)** Fill factors (FFs) of the photovoltaic devices as a function of the light intensity. The FFs increased with the decrease of the light intensity. **(e)** Power densities of the photovoltaic devices as a function of the light intensity. The power changed nearly linearly with the light intensity. **(f)** Overall photoelectric conversion efficiencies (η) of the photovoltaic devices as a function of the light intensity. The overall photoelectric conversion efficiencies of the devices increased with the decrease of the light intensity, which demonstrated the remarkable weak light characteristics of the plasmonic enhanced photovoltaic devices.

References

[S1] Falkovsky, L. A. Optical properties of graphene. *J. Phys.: Conf. Ser.* **129**, 012004 (2008).

[S2] Sachan, R. *et al.* Oxidation-resistant silver nanostructures for ultrastable plasmonic applications. *Adv. Mater.* **25**, 2045–2050 (2013).

[S3] Ferrari, A. C. *et al.* Raman spectrum of graphene and graphene layers. *Phys. Rev. Lett.* **97**, 187401 (2006).

[S4] Zhang, Q. A. *et al.* Seed-mediated synthesis of Ag nanocubes with controllable edge lengths in the range of 30-200 nm and comparison of their optical properties. *J. Am. Chem. Soc.* **132**, 11372−11378 (2010).
